# Assessing the Spatial Distribution of Biodiversity in a Changing Temperature Pattern: The Case of Catalonia, Spain

**DOI:** 10.3390/ijerph16204026

**Published:** 2019-10-21

**Authors:** Diego Varga, Mariona Roigé, Josep Pintó, Marc Saez

**Affiliations:** 1 Research Group on Statistics, Econometrics and Health (GRECS), University of Girona, 17003 Girona, Spain; dievarga@gmail.com; 2 Landscape Analysis and Management Laboratory, University of Girona, 17004 Girona, Spain; josep.pinto@udg.edu; 3 CIBER of Epidemiology and Public Health (CIBERESP), 28029 Madrid, Spain; 4 Bio-protection Research Centre, Lincoln University, Lincoln P.O. Box 85084, New Zealand; mariona.roige.valiente@gmail.com

**Keywords:** biodiversity, catalonia, climate change, geographic range, land use, temperature

## Abstract

The impacts that climate change and land-use dynamics have on biodiversity are already visible in the distribution and behaviour of a large number of species. By using a Bayesian framework, including land-use, meteorological, topography and other variables as explanatory variables, such as distance to roads and urban centres, we modeled a number of species within each cell of a regular lattice for Catalonia, Spain, in the period of 2004 to 2010. We estimated a slight increase in daily maximum temperature and a more significant increase in minimum temperature (a 5-year increase of 0.159 °C in maximum temperature, and an increase of 0.332 °C in minimum temperature). The estimation shows that the total number of species was greater than expected in the cells where land use was not urban—38.4%, in forests and 55.2% in mixed forests. Finally, we observed that most invasive species are found in areas where the minimum temperature is expected to increase. Our study can help with making important recommendations as to where, when and how future threats could affect specie distribution and the kind of planning processes needed for when protected natural areas will be unable to continue to support all the species they were designed to protect.

## 1. Introduction

Climate change represents a significant threat to global biodiversity and natural ecosystem integrity. In the last century, the global average temperatures have risen by 0.7 °C and are predicted to continue rising (IPCC 2013). Future warming in the Mediterranean basin is expected to exceed global rates by 25%, notably with summer warming at a pace 40% higher than the global mean. A global atmospheric temperature increase will probably be accompanied by a reduction in summer precipitation [1]. Climate change will cause potential directional impact on species distribution. Gradual changes that allow ecosystems to slowly adapt. In the IPPC chapter as ecosystems that are adaptive because they are able to change their composition or function in response to changing environmental conditions [2].

Many environmental and biological questions have arisen as a result of this impending transformation. Here, we focus on those related to biodiversity. Losing and moving biological diversity has numerous effects on both natural and social systems, and casts new doubts about conservation planning and policies [3,4,5] as well as the economic and social impact that the loss of some important ecosystem services will have [6,7]. It has been shown in the past that the loss of biodiversity is mainly due to three important factors (and their interaction): (1) changes in land use (fragmentation), (2) the introduction of invasive alien species and (3) global warming [8,9,10,11]. The relationships between these three causes cannot be stated with any certainty because they are highly complex and also because of the well-known fact that relationship does not mean causality [12].

Land-use change is a complex process and, once analysed, can be the input to assess interlinked processes such as climatic variability, land degradation, ecosystem stability, and biodiversity [13]. Human population growth and resource use, mediated by changes in climate, land use, and water use, increasingly impact biodiversity [14,15].

Nevertheless, an interesting fact has been observed, one that is closely related to the abovementioned factors and to the definition that the IPPC gives to “ecological adaptation”. As was widely reported and reviewed in the last IPCC [2] many plant and animal species have shifted their ranges and altered their abundance and seasonal activities in response to the observed climate change that has taken place over the last decades [14,16,17]. When this information is compared to the pale ecological records, the predictions result in large-scale biome shifts and changes in community composition. Although there is strong evidence to believe these changes are occurring, and will keep on doing so, the magnitude of the actual changes is still to be determined and is likely to be very different across geographical areas and ecoregions. However, the differences for every individual species are also considerable, thus leading to the idea that the range shift for each species depends on multiple internal and external traits [18]. Climate change is the main issue on the environmental politics agenda and while it is expected to become a significant driving force behind biodiversity change in the 21st century, it is land use that is projected to remain the foremost impetus of change [19]. Despite climate change and change in land use being key to changes in biodiversity [19,20,21,22,23], the interaction between these two forces is complex and currently not well understood [22,23,24].

It is assumed that Europe will be highly affected by the range shifts and there is the worrying likelihood of the extinction of species [9,25,26]. Some suggest that within Europe, the Mediterranean climate and the grassland ecosystems will experience the greatest proportional change in biodiversity [15,19,27]. Mediterranean-type ecosystems are characterized by cold winters and hot dry summers. Predictions for the area say that a large number of mammals will be endangered and, for Spain and North Africa, the changes are expected to be in terms of loss of species, especially in the protected areas [28]. Not only that, but meta-analyses carried out in 2010 by Gonzalez et al. [29] predict that temperate mixed and boreal conifer forests will experience the greatest vulnerability, and vegetation shift projections suggest potential latitudinal biome shifts of up to 400 km. The effect that temperature and changes in land use has on these correlations has been studied on a local scale.

Biodiversity locations can be associated to their spatial coordinates, the temporal instant, and the corresponding covariates. This association facilitates the representation of a biodiversity as a realization of a spatio-temporal stochastic process. Spatio-temporal clustering of biodiversity might indicate the presence of risk factors which are not evenly distributed in space and time. In fact, what is usually of interest is to assess the association of clustering of biodiversity to spatial and seasonal covariates [30].

In this study, we aimed to test whether good predictions about the range shifts can be made on a local scale, and to test whether the results of our predictions follow the trends of the global ones. We identify areas most susceptible to future biodiversity loss. There are many reasons for doing a region-specific study, but the most important would be the fact that, among biomes, there are large differences as to what the causes of the biodiversity changes are [19], and also because many terrestrial species have small geographic ranges and cannot be accounted for in larger scale analyses [9]. Furthermore, while management policies in Spain undoubtedly depend on the European framework [31], region-specific studies can help local policy makers make decisions based on objective facts, and provide them with species-specific information that potentially provides enormous help to better manage protected areas, as well as a useful mechanism to quickly identify areas of biological and conservation interest. This is currently used to select and design protected areas. Changes in temperature can carry changes in biodiversity by creating opportunities for previously innocuous alien species by enhancing their reproductive capacity, their survival and their competitive power against the native flora and fauna

In our study, we focused on the region of Catalonia (NE Spain). Catalonia is an area rich in a variety of landscapes and environmental conditions that favour a great diversity in vegetation [32]. Also, the area is suitable for bio-geographical approaches thanks to the large amount of reliable species distribution data that is readily available. The novelty of our study is that we use an original statistical method in which we include both the explanatory variables observed and confounders not observed and in which we perform a spatial adjustment, with the objective to evaluate which factors were associated with the biodiversity changes over time and to evaluate the spatial variation of these changes in Catalonia from 2007 to 2011.

## 2. Materials and Methods

### 2.1. Data Setting

We use a spatio-temporal ecological design. The population studied corresponded to The Natural Heritage and Biodiversity Spanish Inventory (NHBSI) [33], which is the main information knowledge tool for supporting the objectives and targets of Spain’s biodiversity policy. It contains biodiversity data with over 30 inventories, catalogues and lists of species for the period of 2007 to 2011. All of this information can be found in the Nature Data Bank [34].

### 2.2. Variables and Information Sources

#### 2.2.1. Response Variables

As the response variable, we considered the total number of species (amphibians, reptiles, birds and mammals) in each of the 10 × 10 km cells, into which the Spanish Inventory of Natural Heritage and Biodiversity divides all of Catalonia [34].

#### 2.2.2. Explanatory Variables

Our explanatory variables of interest were the average of the variations in the maximum and minimum temperatures in each of the cells of one year with respect to the previous year in the period 2007 to 2011. Specifically, the variation on day *k* was calculated first, such as the difference between the value of the temperature (maximum and minimum) and that occurred on the same day in the previous year. Variations were averaged for the whole period considered and they were stratified by season (spring, summer, autumn and winter).

The maximum and minimum temperature data, recorded daily for the period 1 January 2007 to 31 December 2011, from 190 stations throughout the region of Catalonia, were provided by the Weather Area (Meteorological Service of Catalonia). These data were used to estimate the variations (both annual and by season) of maximum and minimum temperatures in each of the cells (further details can be found in [35]).

Also, for each of the cells we included some spatial variables as control variables: (i) topographic variables—slope, aspect, hill shade and elevation [36,37], (ii) proximity to anthropic areas (these five variables were categorized in quintiles, taking the first as a reference category) and (iii) land use (the variable categorized in eight categories, again the first as the reference) [19,38].

Slope (expressed in degrees) was the steepness or degree of incline of a surface. Here, for a particular location, slope was computed as the maximum rate of change in elevation between the location and its surroundings. Aspect was measured clockwise in degrees from 0 to 360. Hill shading is a technique used to visualize terrain as shaded relief by illuminating it with a hypothetical light source. Here, the illumination value for each cell was determined by its orientation to the light source which, in turn, was based on slope and aspect and was also measured in degrees, from 0 to 360. Finally, elevation was considered as elevation above sea level and expressed in meters. To obtain topographic variables (DTM) we have used the MET-15 model, which is a regular grid containing orthometric heights distributed according to a 15 m cell side, and has been created by the Cartographic and Geological Institute of Catalonia [30,39].

The proximity to anthropic areas could be considered as a factor explaining biodiversity loss due to habitat fragmentation. Proximity to anthropic areas was approached through the distances, in metres, from the location of each cell to urban areas, roads and railroads. These distances were constructed by considering a geographical layer in each case. The urban area and road layers were obtained from the Catalan Government’s Department of Territory and Sustainability, through the ICGC.

We also used the land use on Catalonia maps (1:250,000), with classification techniques applied on existing LANDSATMSS images for 1997 and 2002 [40,41]. Additionally, we used orthophoto maps (1:5000) from 2005 to 2007 to create the land use map for 2010 (30 m cell) with an accuracy of over 92% [42]. Specifically, we assigned the land use map just before the date of each wildfire. We assigned, as the land use for each buffer, only the percentage value corresponding to the principal land use of the buffer within. In this paper, we transformed the twenty-two categories, obtained from the ICGC cover map of Catalonia, into eight categories: coniferous forests, dense forests (tree canopy density of 40% and more but less than 70%), fruit trees and berries, artificial non-agricultural vegetated areas, transitional woodland scrub, natural grassland, mixed forests and other (urban, beaches, sand, bare rocks, burnt areas, and water bodies).

### 2.3. Statistical Analysis

We assumed that in each cell the total number of species (being a discrete variable, i.e., a counting variable) follows a Poisson distribution

(1) $$O_{i}\sim Poisson\left(\mu_{i}\right),$$

where *O_i_* denotes the total number of species in the cell *i*.

We are interested in modeling relative risks, which measure the association between the explanatory variables and outcome (the number of species in our case). The relative risk is associated with risk factors by means of spatial ecological regression.

#### Spatial Adjustment

In addition to the explanatory variables, there could be other unobserved variables (unobserved confounders) that could be associated with the dependent variable.

When one has a spatial design (as in our case), the most important source of non-observed confounding is “spatial dependence” or clustering. That is to say, cells that are close in space show more similar behavior than cells that are not close. In fact, this dependence could be the consequence of unobserved confounders that were spatially distributed. To capture the spatial dependency, in the regression we included a structured random effect with a Matérn structure explicitly constructed through the Stochastic Partial Differential Equation approach [43], indexed by the cell. Further, by introducing an additional unstructured random effect into the model, indexed by the cell, we also controlled for the presence of heterogeneity, that is to say, unobserved variables, invariant over time, that are specific to the unit of analysis.

Given the complexity of our model, we preferred to perform inferences using a Bayesian framework. This approach is considered the most suitable to account for model uncertainty, both in the parameters and in the specification of the models. Moreover, only under the Bayesian approach is it possible to model extra variability with relatively sparse data in some cases. Finally, within the Bayesian approach, specifying a hierarchical structure on the (observable) data and (unobservable) parameters, which are all considered as random quantities, is straightforward. In particular, we followed the Integrated Nested Laplace Approximation (INLA) approach [44], within a (pure) Bayesian framework. As is known, in Bayesian analysis the choice of the prior may have a considerable impact on the results. For this reason, we used penalising complexity (PC) priors here [45].

All analyses were performed with the free software R (version 3.4.2) [46] made available through the INLA library [44,47].

## 3. Results

**Figure 1:**
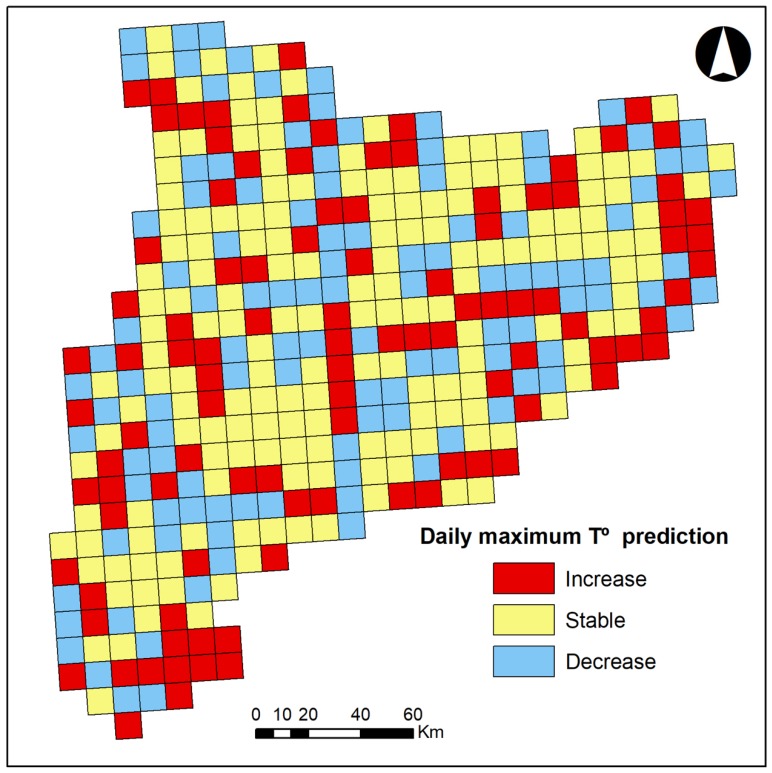

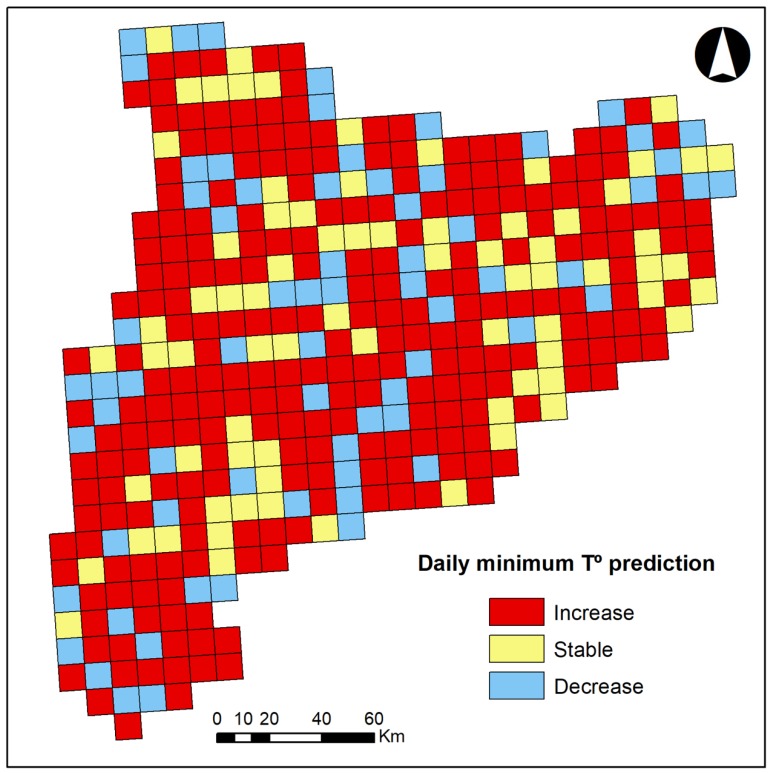
Daily maximum and minimum temperature prediction and latitude variation, with a maximum in 4.619.000 coord Y (ETRS89 31N).

We have estimated a slight increase in daily maximum temperature (0.773%, annualized on average) and a more significant increase in minimum temperature (2.960%, annualized on average) (see Figure 1). These values correspond to a 5-year increase of 0.159 °C (95% credible interval 0.080 °C, 0.242 °C) in maximum temperature, and an increase of 0.332 C (95% credible interval 0.008 °C, 0.635 °C) in minimum temperature. There was an increase in the spatial variability of daily air temperature (estimated standard deviation equals 7.679 °C, maximum temperature, and 8.742 °C, minimum temperature, in 2008), denoting the existence of space–time interactions. In fact, a geographical pattern could be observed in the variations of daily temperature. It seems that the variation of the temperature depended on the latitude, with a maximum in the latitude corresponding to 4.619.000 coord Y (ETRS89 31N).

Furthermore, in our case, the estimated temperature variation was not homogeneous amongst months: This variation has been positive from April to September in maximum temperature and has been positive from May to October in minimum temperature.

The results of the estimation show that the total number of species was greater than expected (relative risk greater than unity) in those cells with some slope (particularly greater than the first quintile, between 0% and 0.37% slope) and with land use other than urban (the predominant category in “others”, that included urban, beaches, sand, bare rocks, burnt areas, and water bodies) (Table A1 in Appendix A). It should be noted that when the slope of the cell was greater than 7.4%, the total number of species in the cell was estimated between 11.8% (95% credibility interval—equivalent to confidence interval—95% CrI 0.04%–24.5%) and 12.9% (95% CrI 0.01%–27.6%) higher than expected. On the other hand, in the cells whose land use was not urban, the total number of species was between 38.4%, in forests, and 55.2%, in mixed forests, higher than expected. However, in all cases the statistical significance was only marginal (the 90% credibility intervals did not contain the unity) and the credibility intervals overlap and therefore were not found statistically different (Table A1 in Appendix A).

In contrast, in those cells closest to inhabited areas and with an orientation of the slope (i.e., aspect) of the fourth quartile, the total number of species was estimated to be lower than expected (i.e., relative risk less than unity; Table A1 in Appendix A). In this sense, when the distance to inhabited areas was less than 180 metres (second quintile) the total number of species was 4.2% fewer than when the distance was between 180 and 1664 m; and 9.5% when the distance was greater than 1664 m (fifth quintile). In addition, when the aspect of the cell was greater than 257.9 m (fourth quartile), the number of species was 6.4% less.

**Figure 2:**
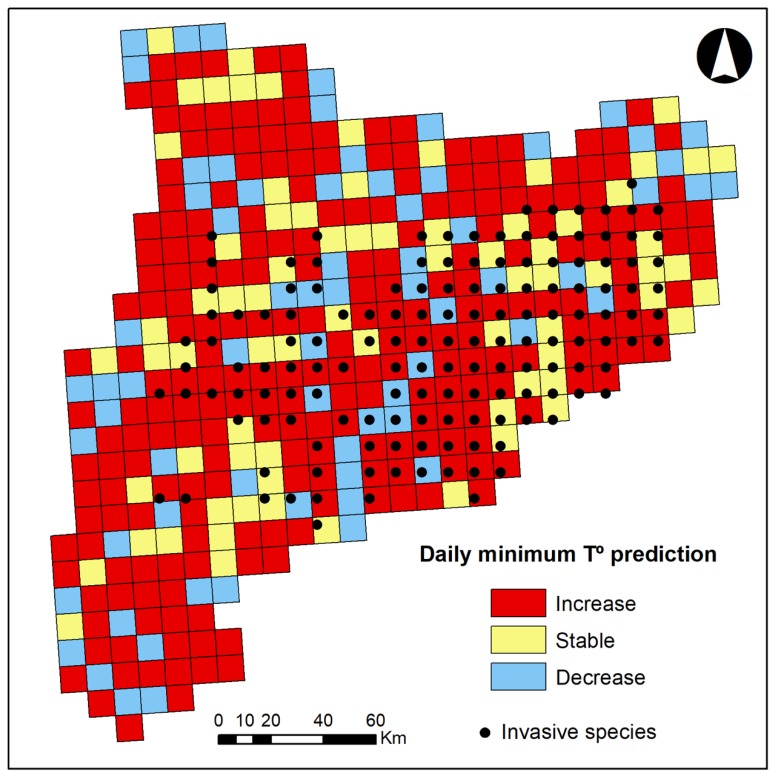
Minimum temperature prediction and invasive species distribution.

We can observe that most invasive species are found in areas where the minimum temperature is expected to increase (Figure 2).

Many studies have shown that different species are colonizing new territories due to the increase in temperature. These species look for higher latitudes where they can find their optimal conditions. This study shows that the latitudinal range where the temperature changes are greatest has been calculated locally. On the other hand, we have verified that protected areas where biodiversity accumulates are becoming areas of biodiversity loss at the expense of other areas that are increasing biodiversity because the species seek their optimum at higher latitudes.

**Figure 3:**
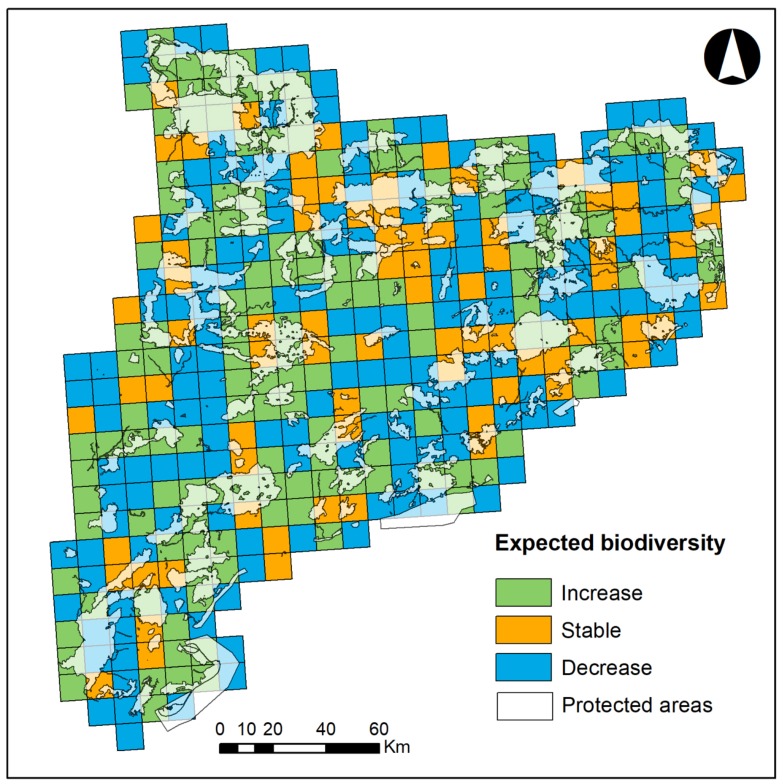
Correspondence between the expected biodiversity and current location of protected areas.

Protected areas are crucial for conserving the remaining biodiversity. We estimated the composition of several well-studied taxonomic groups (amphibians, reptiles, birds and mammals), and although it is observed that even most protected areas will concentrate the greatest biodiversity, we must consider that new spaces will be colonized by new species and that we will find hotspots outside protected areas (Figure 3).

## 4. Discussion

Temperature and land use will lead to broad-scale changes in specie patterns on regional scales. Predictions play an important role in alerting decision-makers to potential future risks and can support the development of management strategies to reduce temperature change impacts on biodiversity.

The establishment of protected areas is traditionally considered indispensable to preserve biodiversity hotspots or areas inhabited by threatened species. The results show that the response of specie richness to the change in temperature could cause a shift between these two areas, i.e., where there is no type of protection measure and invasive species colonize new areas as protected natural areas compete with protected species of great ecological value. Invasive species are defined as a non-native plant, animal or other organism that dominates the new colonized ecosystem. Invasive species can dominate these natural ecosystems by displacing native fauna and flora species. Both situations present a risk for the conservation of biodiversity and hence the study highlights the importance of habitat to habitat heterogeneity in Mediterranean landscapes is as a criterion for landscape planning and for defining management directives to preserve this changing biodiversity.

It is important to have a global view of the biodiversity pattern to design and manage protected spaces and biological corridors, hence having a global view of the biodiversity pattern is fundamental. Climate change often is seen as a future problem that is insignificant in comparison to other pressures such as fragmentation [15,24,38,48] but protected areas are geographically fixed and increasingly isolated by habitat destruction, and are therefore poorly suited to accommodating species range shifts due to climate change.

Biodiversity conservation should be planned on large spatial scales, which means increasing the focus on the landscape matrix. Protected areas need to be connected via corridors or “stepping-stone” patches. A lack of these connectivity infrastructures could have a huge effect on overall biodiversity and the efficiency of protected natural areas (protected areas could be islands within fragmented landscapes). The modeling of the expected biodiversity can improve the location of corridors and can help managers to develop conservation strategies and restoration techniques.

## 5. Conclusions

The ranges of plants and animals are moving in response to recent changes in climate. This study shows that the latitudinal range where the temperature changes, calculated locally, are greatest. On the other hand, we have verified that protected areas where biodiversity accumulates are becoming areas of biodiversity loss at the expense of other areas that are increasing in biodiversity because the species seek their optimum at higher latitudes.

A great conservation challenge would be facilitating the movement of species across the landscape matrix so that they can move to sites that, in the future, provide suitable conditions while ensuring the continued viability of individual protected natural areas.

A new policy of design protected areas has been established to contemplate the effects of climate change. Sufficient evidence exists to show that early implementation of new protected areas is important to reduce the threat that climate change poses to biodiversity. Concepts such as connectivity and corridors are much more frequently proposed as responses to climate change.

## Declaration

We used data from the Nature Data Bank and from the Cartographic and Geological Institute of Catalonia (ICGC). Public and freely accessibles at: http://www.mapama.gob.es/en/biodiversidad/temas/inventarios-nacionales/inventario-espanol-patrimonio-natural-biodiv/sistema-indicadores/descarga-datos-abiertos.aspx; http://www.icgc.cat/en/.

## Appendix A

**Table A1:** Results of the estimation of the models explaining the total number of species (amphibians, reptiles, birds and mammals) in each of the 10 × 10 km cells in Catalonia, 2007–2011.

Variables	Relative Risk	95% Credibility Interval	
Variations in the temperature (°C)			
Maximum temperature	1.275	0.698	2.322
Minimum temperature	0.933	0.493	1.767
Hill shade and elevation [1st Quintile < 161 m]			
2nd Quintile 161–334 m	0.967	0.869	1.074
3rd Quintile 335–608 m	0.945	0.826	1.079
4th Quintile 609–1069 m	0.968	0.825	1.132
5th Quintile 1070–2541 m	0.968	0.801	1.166
Distance to anthropic areas [1st Quintile < 180 m]			
2nd Quintile 180–539 m	0.948	0.867	1.036
3rd Quintile 540–905 m	0.991	0.907	1.082
4th Quintile 906–1664 m	0.976	0.891	1.068
5th Quintile 1665–10170 m	0.905	0.822	0.997
Slope [1st Quintile < 0.37%]			
2nd Quintile	1.147	1.047	1.255
3rd Quintile	1.056	0.947	1.178
4th Quintile 7.4%–11.0%	1.118	1.004	1.245
5th Quintile 11.0%–24.4%	1.129	1.001	1.276
Aspect [1st Quartile < 93.0]			
2nd Quartile 93.0–174.0	1.025	0.941	1.115
3rd Quartile 174.1–257.0	0.993	0.919	1.072
4th Quartile 257.1–357.0	0.936	0.848	1.032
Land use [Other]			
Coniferous forests			
1997 Cover map	0.948	0.000	51,87,048.404
2002 Cover map	1.075	0.000	5,887,435.884
2010 Cover map	1.486	0.881	2.512
Dense forest			
1997 Cover map	1.077	0.000	5,897,531.078
2002 Cover map	0.914	0.000	5,005,564.082
2010 Cover map	1.384	0.824	2.330
Fruit trees and berries			
1997 Cover map	1.032	0.000	5,647,790.248
2002 Cover map	1.000	0.000	5,474,699.312
2010 Cover map	1.420	0.853	2.366
Artificial, non-agricultural vegetated			
1997 Cover map	0.911	0.000	4,993,598.489
2002 Cover map	1.214	0.000	6,652,178.514
2010 Cover map	1.266	0,000	6,928,848.938
Transitional woodland scrub			
1997 Cover map	0.988	0.000	5,404,510.383
2002 Cover map	1.061	0.000	5,808,358.914
2010 Cover map	1.448	0.863	2.433
Natural grassland			
1997 Cover map	1.256	0.000	6,905,193.758
2002 Cover map	0.977	0.000	5,373,630.141
2010 Cover map	1.276	0.757	2.152
Mixed forest			
1997 Cover map	1.418	0.000	7,762,645.290
2002 Cover map	1.004	0.000	5,502,696.848
2010 Cover map	1.522	0.905	2.567

[Reference category between brackets]. Shaded in grey 95% Credibility interval did not contain 1, shaded in yellow 90% Credibility interval did not contain 1.

Relative risk is interpreted as follows (RR − 1) × 100. Thus, for example, when the slope of the cell was greater than 7.4% and less than 18% (fourth quartile), the total number of species in the cell was estimated at 11.8% ((1118 − 1) × 100) higher than expected.
